# Lipoprotein (a) level as a risk factor for stroke and its subtype: A systematic review and meta-analysis

**DOI:** 10.1038/s41598-021-95141-0

**Published:** 2021-08-02

**Authors:** Pradeep Kumar, Priyanka Swarnkar, Shubham Misra, Manabesh Nath

**Affiliations:** Department of Neurology, All India Institute of Medical Sciences, New Delhi, 110029 India

**Keywords:** Neuroscience, Biomarkers

## Abstract

The role of lipoprotein-A [Lp (a)] as a risk factor for stroke is less well documented than for coronary heart disease. Hence, we conducted a systematic review and meta-analysis for the published observational studies in order to investigate the association of Lp (a) levels with the risk of stroke and its subtypes. In our meta-analysis, 41 studies involving 7874 ischemic stroke (IS) patients and 32,138 controls; 13 studies for the IS subtypes based on TOAST classification and 7 studies with 871 Intracerebral hemorrhage (ICH) cases and 2865 control subjects were included. A significant association between increased levels of Lp (a) and risk of IS as compared to control subjects was observed (standardized mean difference (SMD) 0.76; 95% confidence interval (CIs) 0.53–0.99). Lp (a) levels were also found to be significantly associated with the risk of large artery atherosclerosis (LAA) subtype of IS (SMD 0.68; 95% CI 0.01–1.34) as well as significantly associated with the risk of ICH (SMD 0.65; 95% CI 0.13–1.17) as compared to controls. Increased Lp (a) levels could be considered as a predictive marker for identifying individuals who are at risk of developing IS, LAA and ICH.

## Introduction

Stroke is reported as the most common cause of long term disability and the second most leading cause of death worldwide^1^. Almost 80% of strokes are ischemic stroke (IS) and 15–20% are haemorrhagic stroke (HS) in origin^2,3^. According to the Trial of Org 10172 in Acute Stroke Treatment (TOAST) classification; IS has been categorised according to the presumed etiological mechanism into five groups: large artery atherosclerosis (LAA), small vessel disease (SVD), cardioembolic disease (CE), other determined etiology (ODE), and undetermined etiology (UDE)^4^.

Lipoprotein (a) or Lp (a) is a lipoprotein moiety that consists of core lipoprotein molecule, containing apolipoprotein B (apo-B100), to which a glycoprotein of variable molecular weight, apolipoprotein (a) [apo(a)], is covalently attached via a cysteine-cysteine disulfide bond^5,6^. By binding LDL, calcium, and other components into an atherosclerotic plaque on the walls of blood arteries, Lp (a) is hypothesised to speed up the development of atherosclerosis^7^. The LPA gene regulates the variation in Lp (a) plasma concentrations genetically, ranging from 36% in the PROCARDIS^8^ consortium to 70–90% in genome-wide association studies, with larger apo(a) isoforms related with lower values of Lp (a)^9,10^. The concentrations of Lp (a) range from 0.1 mg/dl to more than 200 mg/dl^11^.

On a cellular level, apo(a) undergoes post-translational changes in the endoplasmic reticulum as a secretory protein (ER). The length of time it takes to modify larger apo(a) isoforms is determined by the size of the apo(a) isoform. As a result, larger apo(a) molecules are produced at a slower rate per unit of time, resulting in lower Lp (a) plasma concentrations^6,12^. Plasma Lp (a) concentrations appear to be regulated by synthesis rather than catabolism, according to kinetic studies. Concentration and pathological responses may be influenced by apo(a) sequence polymorphisms. Lp (a)/apo(a) functions may also be affected by changes in circulating Lp (a). Importantly, the relevance of apo(a) in cardiovascular diseases (CVD s) and peripheral vascular disorders, as well as its physiological function, remain unknown, and there is no effective therapeutic option for decreasing increased Lp levels (a)^11,13–15^.

Other large, population-based cohort studies on stroke have produced mixed results, with some research associating raised Lp (a) levels to a higher incidence of IS^16–19^, while others have found no link^20–22^. This could be due to a lack of discrimination across incidence stroke subtypes^22^, as well as ethnic or other disparities in cohort composition. A growing number of epidemiological studies have found a link between dyslipidemia and atherosclerosis-related stroke. Indeed, the lipid metabolism of different stroke types and IS subtypes differs dramatically^23–26^. Two previously published meta-analyses^27,28^ had confirmed that elevated Lp (a) is an independent risk factor for IS, however, IS subtypes based on TOAST classification as well as HS remains to be explored further. Hence, we conducted a systematic review and meta-analysis for the published observational studies in order to investigate the association of Lp (a) levels with the risk of stroke and its subtypes.

## Methods

### Search strategy

This systematic literature review was performed using the guidelines of the PRISMA statement (Preferred Reporting Items for Systematic Reviews and Meta-analyses)^29^. A comprehensive search for all the published articles was performed in electronic databases including PubMed, EMBASE, Cochrane Library, Trip Databases, Worldwide Science, and Google Scholar from 01st January 1950 to 30th April 2020. Following search terms: ‘Lipoprotein (a)’ OR ‘Lp (a) Levels’ OR ‘Lipid Biomarkers’ AND ‘Stroke’ OR ‘Subtypes’ OR ‘TOAST Classification’ OR ‘Ischemic Stroke’ AND ‘Haemorrhagic Stroke’ OR Intracerebral Haemorrhage’ OR ‘ICH’ AND ‘Cerebrovascular Disease’ AND ‘cerebral infarction’ were used. Reference lists of the selected studies were also searched manually to obtain any additional eligible studies on human subjects. No restrictions related to language, sex and publication year was applied.

### Eligibility criteria

#### Inclusion criteria

(1) Observational studies including case–control, nested case–control, cross sectional and cohort design investigating the association of Lp (a) levels with the risk of stroke or stroke types or IS sub-types based on TOAST classification compared to control subjects; (2) studies with clinically confirmed diagnosis of stroke (ischemic or haemorrhagic) using CT or MRI scans; (3) patients aged ≥ 18 years (adult population); (4) studies reporting numbers for patients and control groups as well as raw values for Lp (a) levels.

#### Exclusion criteria

(1) Duplicates, case reports, case series, systematic reviews, conference abstracts, preprints and editorials; (2) Studies not reporting relevant outcomes; (3) Unavailability of full-texts.

### Risk of bias in individual studies

The risk of bias was assessed by Newcastle–Ottawa Scale (NOS) for quality assessment of all the included studies in the meta-analysis^30^. The assessment criteria involving NOS uses three broad criteria viz. selection, comparability and exposure. Selection criteria defines and analyses the cases and control subjects included in the study, comparability defines the matching or comparison of cases and control subjects for better empirical investigation and exposure determines whether the study was conducted in a blinded or unbiased manner along with the response of the subjects. Publication bias was assessed using Begg’s and Egger’s funnel plot analysis^31,32^.

### Data extraction

All relevant studies were analysed separately by two reviewers (PK and PS) based on the inclusion criteria listed above. The analysis was done first at the title and abstract level and then at the full-text level. Any disagreement was resolved by discussion with a third reviewer. Following data were extracted from the studies which included: First Author’s Name, Published Year, Ethnicity, Country, Study Design, Number of Cases and Controls, Mean Age, mean and standard deviation values of biochemical parameters including Lp (a), methods of Lp (a) assay and follow up duration. Data was extracted independently by two authors (PK and PS) using a standardized extraction table. Lp (a) concentrations were reported with different units in the included studies and were converted to similar units for analysis purpose using online unit conversion tools (http://unitslab.com/node/85).

### Statistical analysis

A random or fixed effect model was used to calculate the pooled Standardized Mean Difference (SMD) or Odds Ratio (OR) with 95% confidence interval (CI). Heterogeneity was calculated with the *I*^2^ statistic and was adjusted by subgroup analysis followed by meta-regression using the quality score of the included studies. The heterogeneity was considered as significant in case of *I*^2^ more than 50% for which random-effects model was applied, on the other hand, if *I*^2^ was less than 50%, then fixed-effect model was applied. A sensitivity analysis was performed by sequentially omitting a single study in each turn, to validate the pooled observed effect. Tests were considered statistically significant at a p-value less than 0.05. Data were analyzed using STATA, version 13.0 (Stata Statistical Software, Release 13; StataCorp LP, College Station, TX).

### Statement of ethics

Ethical approval was not required for this manuscript as it was a systematic review and meta-analysis done by using the existing published data and the research was not directly conducted in any human subjects.

## Results

**Figure 1:**
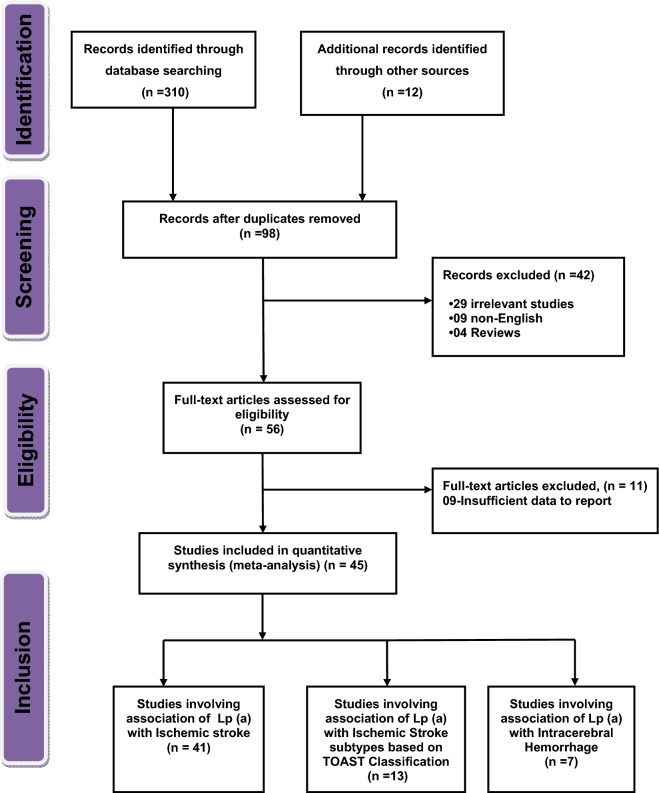
Flow diagram for the selection of studies and specific reasons for exclusion from the present meta-analysis.

Figure 1 represents the PRISMA flow diagram listing the detailed reasons for exclusion and inclusion of studies in our systematic review and meta-analysis. Initially, a total of 322 studies were identified after searching in six different databases. PRISMA checklist has been provided in the supplementary table (Table S1). After removing duplicate articles, 98 articles were found and on further exclusion, a total of 56 full text articles were reviewed for eligibility and finally 45 studies were included in the systematic review and meta-analysis. The baseline characteristics of all the included 45 studies (41 studies for investigating the association of LP (a) with the risk of IS; 13 studies for IS subtypes and 7 studies for ICH) are given in Tables 1, 2 and 3.

**Table 1:** Baseline characteristics of studies included in the systematic review and meta-analysis for the relationship between serum Lp (a) levels and risk of ischemic stroke.

S. no.	Author name and year	Ethnicity	Study design	Source of control	Sample size (IS/control)	IS age (mean ± SD)	Control age (mean ± SD)	Matching criteria	LPA assay method	LPA cut off value	LPA timepoint	Follow up duration	NOS quality score
1.	Shintani et al., 1993^33^	Asian	Case–control study	HB	54/81	62 ± 8.1	61.1 ± 8.6	NA	ELISA	≥ 42.6 mg/dl	4 weeks	NA	5
2.	More et al., 2017^34^	Asian	Case–control study	HB	100/50	NA	NA	NA	NA	≥ 30 mg/dl	NA	NA	4
3.	Albala et al., 2010^35^	Caucasian	Case–control study	PB	317/413	69.7 ± 12.3	69.7 ± 11.7	Age, sex and race/ethnicity	Immunonephelometric procedure	≤ 30 mg/dl	Within 72 h	NA	6
4.	Kiechl et al., 2007^36^	Caucasian	Prospective cohort study	PB	82/683	70.2 ± 10.3	61.8 ± 10.9	Age and sex	ELISA	≥ 24 mg/dl	NA		7
5.	Christopher et al., 1996^37^	Asian	Case–control study	HB	50/50	27 ± 5	27 ± 5	Age, sex and socioeconomic status	ELISA	NA	NA	NA	5
6.	Fu et al., 2020^38^	Asian	Case–control study	HB	1953/1953	62.3 ± 11.8	59.9 ± 11.1	Age and sex	Latex agglutination turbidimetric method	23.2 mg/dl	NA	NA	7
7.	Dhamija et al., 2009^39^	Asian	Case–control study	HB	66/72	54.43 ± 13	54.4 ± 13	Age and sex	Immunoturbidimetric immunoassay	≤ 30 mg/dl	Within 12 h	NA	6
8.	Shao-yi-Li et al., 2014^40^	Asian	Prospective cohort study	PB	181/120	63 ± 4.6	62.5 ± 5.7	Age and sex	Immunoprecipitation techniques	≥ 30 mg/dl	Within 24 h	NA	7
9.	Milionis et al., 2005^41^	Caucasian	Case–control study	PB	163/166	77.6 ± 4.8	77.7 ± 4.8	Age and sex	Immunoprecipitation techniques	≥ 30 mg/dl	Within 24 h	No	7
10.	Peng et al., 1999^42^	Asian	Case–control study	HB	90/90	62.6 ± 8.9	63.1 ± 8.3	NA	ELISA	NA	Within 24 h	No	4
11.	Jurgens et al., 1995^43^	Caucasian	Case–control study	HB	42/288	51.4 ± 7.2	51 ± 7.1	NA	ELISA	20 mg/dl	Within 48 h	NA	7
12.	Ridker et al., 1995^20^	Caucasian	Nested case–control study	PB	198/198	62.5 ± 5	62.1 ± 5	Age, sex and smoking	NA	19.68 mg/dl	NA		7
13.	Rigal et al., 2006^44^	Caucasian	Case–control study	PB	100/100	45.3 ± 7.7	45.1 ± 6.8	Age and sex	Immunoturbidimetric method	30 mg/dl	Within 4 days	NA	6
14.	Sun et al., 2003^45^	Asian	Case–control study	PB	1326/1817	61.1 ± 9.2	59.6 ± 8.5	NA	ELISA	NA	Within 6 weeks	NA	7
15.	Tascilar et al., 2008^46^	Caucasian	Case–control study	PB	85/77	61.6 ± 13.5	54.7 ± 8.4	NA	Latex agglutination assay	NA	NA	NA	5
16.	Zenker et al., 1986^47^	Caucasian	Case–control study	HB	46/37	53.6 ± 9.7	54.4 ± 7.7	NA	Electro immunoassay	NA	NA	NA	4
17.	Botet et al., 1992^48^	Caucasian	Case–control study	HB	100/100	64.4 ± 6	64.4 ± 6	Age	Electro immunoassay	NA	NA	NA	4
18.	Glader et al., 1999^21^	Caucasian	Case–control study	PB	101/201	55.6 ± 6.9	55.6 ± 6.8	Age and sex	ELISA	30 mg/dl	NA	NA	4
19.	Poitrine et al., 2010^49^	Caucasian	Prospective cohort study	PB	98/8978	55.6 ± 3	54.8 ± 2.8	NA	Selective bi-site immunoenzymatic assay	NA	Within 12 h		7
20.	Albucher et al., 2000^50^	Caucasian	Case–control study	PB	94/111	35.8 ± 8.2	35.8 ± 8.2	Age	Rocket immunoelectrodiffusion	NA	NA	NA	5
21.	Markus et al., 1997^51^	Caucasian	Case–control study	HB	164/91	66.1 ± 9.8	64.6 ± 8.2	NA	ELISA	40 mg/dl	NA	NA	6
22.	Alfthan et al., 1994^52^	Caucasian	Prospective cohort study	PB	74/269	54 ± 4	54 ± 4	NA	Two-site immunoradiometric method	NA	NA		5
23.	Chakraborty et al., 2013^53^	Asian	Case–control study	HB	100/120	54 ± 10.9	52.5 ± 9.8	Age and sex	Immunoturbidimetric method	NA	At 1, 7 days, 3 and 6 months		6
24.	Jones et al., 2007^54^	Caucasian	Case–control study	PB	184/230	71.9 ± 10	70.3 ± 6.9	NA	ELISA	> 45 nmol/L	NA	NA	7
25.	Jones et al., 2009^55^	Caucasian	Case–control study	PB	245/439	71.4 ± 10.5	68.8 ± 6.6	NA	ELISA	NA	NA	NA	6
26.	Denti et al., 2003^56^	Caucasian	Case–control study	HB	79/98	82.9 ± 7.4	82.9 ± 7.4	Age and sex	ELISA	NA	Within 48 h	NA	5
27.	Hiraga et al., 1996^57^	Asian	Case–control study	HB	83/39	67.6 ± 10.5	65.3 ± 6.8	NA	Latex immunosorbent assay	NA	NA	NA	4
28.	Pena-Diaz et l., 2003^58^	Caucasian	Case–control study	HB	52/91	53.4 ± 10.5	40.2 ± 13.1	NA	Immunonephelometric method	> 22.45 mg/dl	NA	NA	4
29.	Karttunen et al., 2002^59^	Caucasian	Case–control study	PB	46/104	41.5 ± 3.1	43.7 ± 3.2	NA	ELISA	NA	NA	NA	5
30.	Kario et al., 1994^60^	Asian	Case–control study	PB	31/50	83 ± 5	84 ± 5	NA	ELISA	> 30 mg/dl	Within 4 days	NA	4
31.	Ma Lijuan et al., 2013^61^	Asian	Case–control study	HB	124/64	60.6 ± 12.1	62 ± 9.1	NA	Sandwich ELISA	NA	Within 12 h	NA	6
32.	Murai et al.,, 1985^62^	Asian	Case–control study	HB	156/99	64.8 ± 9	61.5 ± 13.4	Age	Single radial immunodiffusion method	17 mg/dl	NA	NA	4
33.	Lindgren A et al., 1992^63^	Caucasian	Case–control study	PB	119/159	70.7 ± 9.1	60 ± 11.5	Age	Radioimmunoassay	NA	NA	NA	5
34.	Kooten et al., 1996^64^	Caucasian	Case–control study	HB	119/274	66.3 ± 15.4	50.2 ± 7.4	NA	Two-site immunoradiometric assay	NA	NA	NA	6
35.	Peynet et al., 1999^65^	Caucasian	Case–control study	PB	90/84	37.4 ± 8.7	37.4 ± 8.7	Age and sex	Immunonephelometric assay	NA	After 3 months of stroke	NA	6
36.	Petersen et al., 2007^66^	Caucasian	Case–control study	HB	253/63	63 ± 14	60.2 ± 10.6	Age and sex	Double-antibody ELISA	30 mg/dl	NA	NA	6
37.	Saito et al., 1997^67^	Asian	Case–control study	HB	118/95	71 ± 10		NA	Sandwich ELISA	NA	NA	NA	4
38.	Santos-silva et al., 2002^68^	Caucasian	Case–control study	HB	50/29	20–79		Age	Electro immunodiffusion	NA	NA	NA	4
39.	Seki et al., 1997^69^	Asian	Case–control study	HB	64/37	72.1 ± 8.4	61 ± 20	NA	ELISA	NA	NA	NA	5
40.	Schreiner et al., 1994 (Black)^70^	Caucasian	Prospective cohort study	PB	324/14,818	56.6 ± 6	53 ± 6	NA	ELISA	30 mg/dl	NA	NA	5
41.	Zhang et al., 2013^71^	Asian	Case–control study	HB	153/100	63 ± 12.7	63 ± 12.7	Age and sex	Immunoturbidimetric method	NA	NA	NA	6

**Table 2:** Baseline characteristics of studies included in the systematic review and meta-analysis for the relationship between serum Lp(a) levels and risk of ischemic stroke subtypes based on TOAST classification.

S. no.	Author name and year	Ethnicity	Study design	Sample size (IS)	Sample size (LAA)	Sample size (SVD)	Sample size (CE)	Sample size (UDE)	Sample size (ODE)	Sample size (control)	LPA assay method	LPA cut off value	LPA timepoint	Follow up duration	NOS quality score
1.	Shintani et al., 1993^33^	Asian	Case–control study	45	9	34	NA	NA	NA	81	ELISA	≥ 42.6 mg/dl	Within 4 weeks		5
2.	Tang et al., 2019^72^	Asian	Retrospective cohort study	226	119	107	NA	NA	NA	NA	Immunoturbidimetry	30 mg/dl	Within 2 h	NA	7
3.	Cerrato et al., 2002^73^	Caucasian	Prospective cohort study	202	119	83	NA	NA	NA	NA	NA	NA	Within 3 months	NA	6
4.	Sun et al., 2003^45^	Asian	Case–control study	1326	809	517	NA	NA	NA	1817	ELISA		Within 6 weeks		7
5.	Botet et al., 1992^48^	Caucasian	Case–control study	76	48	28	NA	NA	NA	100	Electro immunoassay	NA	NA	NA	5
6.	Markus et al., 1997^51^	Caucasian	Case–control study	163	49	37	62		15	91	ELISA	40 mg/dl	NA	NA	5
7.	Chakraborty et al., 2013^53^	Asian	Case–control study	100	35	21	19	22	3	120	Immunoturbidimetric method		At 1, 7 days, 3 and 6 months	NA	6
8.	Lindgren et al., 1992^63^	Caucasian	Case–control study	119	NA	41	33	35	10	159	Radioimmunoassay	NA	NA	NA	5
9.	Slowik et al., 2002^74^	Caucasian	Case–control study	71	30	41	NA	NA	NA	30	Immunonephelometric assay	> 30 mg/ml	Within 8 months	NA	4
10.	Kooten et al., 1996^64^	Caucasian	Case–control study	119	71	48	20	12		274	Two-site immunoradiometric assay	NA	NA	NA	6
11.	Petersen et al., 2007^66^	Caucasian	Case–control study	254	71	53	62	51	17	63	Double-antibody ELISA	30 mg/dl	NA	NA	6
12.	Saito et al., 1997^67^	Asian	Case–control study	118	13	35	21	17		95	Sandwich ELISA	NA	NA	NA	5
13.	Yokohawa et al., 2008^75^	Asian	Cross-sectional study	161	87	55	19	NA	NA	NA	ELISA	NA	NA	NA	4

**Table 3:** Baseline characteristics of studies included in the systematic review and meta-analysis for the relationship between serum Lp(a) levels and risk of intracerebral haemorrhage (ICH).

S. no.	Author name and year	Ethnicity	Study design	Sample size (ICH/control)	ICH age, years	Control age, years	Source of control	Matching criteria	LPA assay method	LPA cut -off value	LPA assay timepoint	Follow up duration	NOS quality score
1.	Fu et al., 2020^38^	Asian	Case–control study	196/392	57.9 ± 13.7	57.9 ± 13.7	HB	Age and sex	Latex agglutination turbidimetric method	23.2 mg/dl	NA	NA	7
2.	Sun et al., 2003^45^	Asian	Case–control study	499/1817	58.2 ± 9.7	59.6 ± 8.5	PB	NA	ELISA	Within 6 weeks	NA	NA	7
3.	Pena-Diaz et al., 2003^58^	Caucasian	Case–control study	105/91	62.5 ± 10.6	40.2 ± 13.1	HB	NA	Immunonephelometric method	> 22.45 mg/dl	NA	NA	6
4.	Lindgren et al., 1992^63^	Caucasian	Case–control study	12/159	68.9 ± 11.6	60 ± 11.5	PB	Age	Radioimmunoassay	NA	NA	NA	5
5.	Kooten et al., 1996^64^	Caucasian	Case–Control Study	21/274		50.2 ± 7.4	HB	NA	Two-site immunoradiometric assay	NA	NA	NA	4
6	Saito et al., 1997^67^	Asian	Case–control study	32/95	64 ± 11		HB	NA	Sandwich ELISA	NA	NA	NA	4
7	Seki et al.,1997^69^	Asian	Case–control study	64/37	62 ± 9	61 ± 20	HB	NA	ELISA	NA	NA	NA	5

### Characteristics of included studies for ischemic stroke

Out of 45 studies, 41 studies investigated the association of Lp (a) with the risk of IS as compared to control subjects with a total 7874 IS cases and 32,138 control subjects^20,21,33–71^. Thirty-five studies were case–control, one was nested case–control and five were population-based cohort studies. The publication years of the studies included in our meta-analysis ranged from 1985 to 2020. The studies were divided into two groups of populations based on ethnicity; 25 studies were conducted in Caucasian population and 16 studies were in Asian population. The sample size for IS cases ranged from 31 to 1953. Twenty-two studies used hospital-based (HB) source of control and nineteen studies used population-based (PB) source of control. The quality score was high in nine articles, medium in 22 articles and low in 10 articles. The detailed quality scores (NOS scores) of the included studies ranging from low to high are represented in supplementary tables (Table S2).

ELISA technique was found to be most common methods for LPA assay and reported in 18 studies, follow-up duration and LPA timepoints were reported in limited studies. No information was available for the stroke patients undergoing any LPA treatments in all the included studies in our meta-analysis. Only 11 studies^21,35,38,40–42,44,45,52,54,55^ reported calculated OR with 95% CI for the association of Lp (a) with the risk of IS as compared to control subjects; which have been used directly for estimating pooled ORs with 95% CI.

### Characteristics of included studies for Ischemic Stroke subtypes

Thirteen studies^33,45,48,51,53,63,64,66,67,72–75^ reported the association of Lp (a) with the risk of IS subtypes based on TOAST classification. The publication years ranged from 1992 to 2019. Out of 13 studies, eight studies reported data for LAA subtypes^33,45,48,51,53,64,66,74^; nine studies for SVD subtypes^33,45,48,51,53,63,64,66,74^ and five studies for CE subtypes^51,53,63,64,66^ with control subjects. Seven studies were conducted in Caucasian population and six studies were in Asian population. The sample size for IS subtypes ranged from 09 to 1809. Ten studies were case–control, one was cross-sectional and two were population-based cohort studies. The quality score was medium in nine studies, high in two studies and low in two studies as represented in supplementary tables (Table S3).

### Characteristics of included studies for Intracerebral hemorrhage

Only seven studies involving 871 Intracerebral hemorrhage (ICH) cases and 2865 control subjects were identified for the association of Lp (a) levels with the risk of ICH as compared to control subjects^38,45,58,63,64,67,69^. The publication years ranged from 1992 to 2020. Out of seven studies, three studies were conducted in Caucasian population and four studies were in Asian population. The sample size for ICH ranged from 06 to 499. Six studies were case–control and one was a nested case–control study. The quality score was medium in three studies, high in two studies and low in two studies as represented in supplementary tables (Table S4).

### Association of Lp (a) levels with risk of ischemic stroke

**Figure 2:**
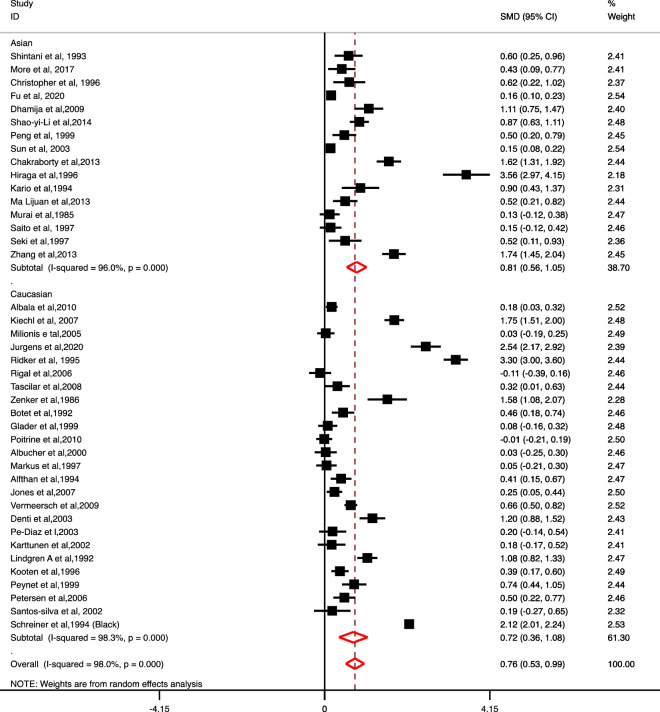
Forest plot for the association of Lp (a) level with the risk of Ischemic stroke vs. control based on ethnicity.

A significant association between increased levels of Lp (a) and risk of IS as compared to control subjects was observed (SMD 0.76; 95% CI 0.53–0.99). A subgroup analysis based on ethnicity also showed a significant association between increased levels of Lp (a) and the risk of IS in 16 Asian (SMD 0.81; 95% CI 0.56–1.05) as well as 25 Caucasian studies (SMD 0.72; 95% CI 0.36–1.08) respectively (Fig. 2).

Based on study design, further subgroup analysis also showed a significant association between increased levels of Lp (a) and the risk of IS as compared to control groups in 35 case–control studies (SMD 0.64; 95% CI 0.48–0.80) and one nested case–control study (SMD 3.30; 95% CI 3.00–3.60) (Table 4). However, we did not observe any significant association between Lp (a) levels and risk of IS in the subgroup consisting of five prospective cohort studies (SMD 0.96; 95% CI − 0.01 to 1.93).

**Table 4:** Summary of findings for the association of LP (a) with the risk of stroke types and subtypes.

Variable	IS vs. control (no. of studies = 41)			LAA vs. control (no. of studies = 08)			SVD vs. control (no. of studies = 09)			CE vs. control (no. of studies = 05)			ICH vs. control (no. of studies = 07)		
	SMD (95% CI)	I^2^ (%)	p-value	SMD (95% CI)	I^2^ (%)	p-value	SMD (95% CI)	I^2^ (%)	p-value	SMD (95% CI)	I^2^ (%)	p-value	SMD (95% CI)	I^2^ (%)	p-value
**Based on study design**															
Case–control studies	**0.64 (0.48 to 0.80)**	**94.7**	**< 0.0001**	**0.32 (0.00 to 0.64)**	**89.1**	**< 0.0001**	− 0.06 (− 0.46 to 0.34)	93	< 0.0001	0.05 (− 1.11 to − 1.21)	97.2	< 0.0001	0.39 (− 0.07 to 0.84)	94.5	< 0.0001
Nested case control studies	**3.30 (3.00 to 3.60)**	–	–	–	–	–	–	–	–	–	–	–	2.24 (1.76 to 2.72)	–	–
Prospective cohort studies	0.96 (− 0.01 to 1.93)	99.2	< 0.0001	–	–	–	–	–	–	–	–	–	–	–	–
**Overall**	**0.76 (0.53 to 0.99)**	**98**	**< 0.0001**	**0.32 (0.00 to 0.64)**	**89.1**	**< 0.0001**	− 0.06 (− 0.46 to 0.34)	93	< 0.0001	0.05 (− 1.11 to − 1.21)	97.2	< 0.0001	**0.65 (0.13 to 1.17)**	**96**	**< 0.0001**
**Based on ethnicity**															
Asian	**0.81 (0.56 to 1.05)**	**96**	**< 0.0001**	0.08 (− 0.22 to 0.39)	59.1	0.087	− 0.56 (− 1.98 to 0.85)	98	< 0.0001	− 2.58 (− 3.15 to − 2.00)	–	–	0.41 (− 0.24 to − 1.06)	96.6	< 0.0001
Caucasian	**0.72 (0.36 to 1.08)**	**98.3**	**< 0.0001**	0.45 (− 0.10 to 0.99)	98.1	< 0.0001	0.16 (− 0.08 to 0.40)	57.1	0.04	0.08 (− 0.18 to 1.54)	94.2	< 0.0001	0.98 (− 0.32 to 2.28)	94.2	< 0.0001
Overall	**0.76 (0.53 to 0.99)**	**98**	**< 0.0001**	**0.32 (0.00 to 0.64)**	**89.1**	**< 0.0001**	− 0.06 (− 0.46 to 0.34)	93	< 0.0001	0.05 (− 1.11 to − 1.21)	97.2	< 0.0001	**0.65 (0.13 to 1.17)**	**96**	**< 0.0001**
**Based on NOS quality score**															
High	**0.99 (0.53 to 1.44)**	**98.9**	**< 0.0001**	**0.16 (0.08 to 0.25)**	–	–	**0.14 (0.04 to 0.24)**	–	–	–	–	–	0.61 (− 0.34 to 1.56)	98.8	< 0.0001
Medium	**0.66 (0.32 to 1.00)**	**97.6**	**< 0.0001**	0.99 (− 0.23 to 0.74)	89.8	< 0.0001	− 0.12 (− 0.79 to 0.56)	94.7	< 0.0001	0.05 (− 1.11 to − 1.21)	97.2	< 0.0001	**0.27 (0.03 to 0.52)**	**0**	**0.48**
Low	**0.77 (0.33 to 1.21)**	**93.8**	**< 0.0001**	**0.93 (0.55 to 1.32)**	**–**	**–**	0.00 (− 0.33 to 0.33)	–	–	–	–	–	1.06 (− 1.27 to 3.38)	98.2	< 0.0001
Overall	**0.76 (0.53 to 0.99)**	**98**	**< 0.0001**	**0.32 (0.00 to 0.64)**	**89.1**	**< 0.0001**	− 0.06 (− 0.46 to 0.34)	93	< 0.0001	0.05 (− 1.11 to − 1.21)	97.2	< 0.0001	**0.65 (0.13 to 1.17)**	**96**	**< 0.0001**

*SMD* standardized mean difference, *CI* confidence interval, *IS* ischemic stroke, *LAA* large artery atherosclerosis, *SVD* small vessel disease, *CE* cardioembolism, *ICH* intracerebral haemorrhage.

Bold values of OR represent statistically significant results (p-value < 0.05).

On the basis of NOS quality grading, we also observed a significant association between increased levels of Lp (a) and the risk of IS as compared to control groups in high-quality studies (SMD 0.99; 95% CI 0.53–1.44); medium (SMD: 0.66; 95% CI 0.32–1.00) and low-quality studies (SMD 0.77; 95% CI 0.33–1.21) (Table 4).

**Figure 3:**
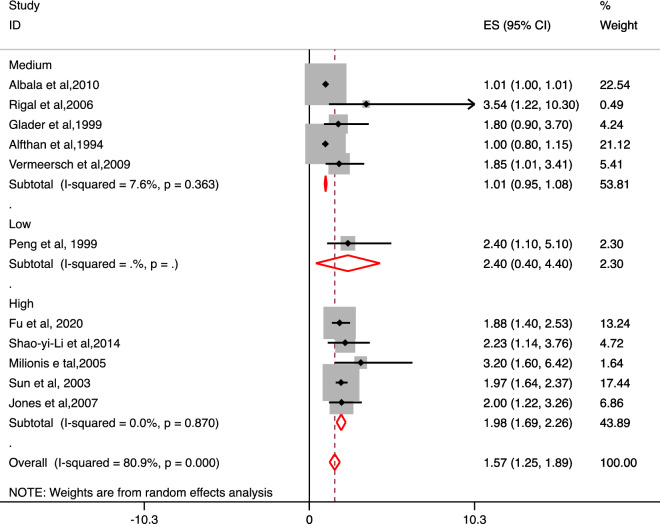
Forest plot for the association of Lp (a) level with the risk of Ischemic stroke vs. control for the reported Odds ratio in the included studies based on NOS quality grading.

Based on extraction of reported calculated OR values directly from eleven studies, we observed an overall a significant association of increased levels of Lp (a) with the risk of IS as compared to control groups (OR 1.57; 95% CI 1.25–1.89). Based on ethnicity, a significant association of increased levels of Lp (a) with the risk of IS as compared to control groups was observed for Asian population (OR 1.97; 95% CI 1.67–2.26) but not for Caucasian population (OR 1.10; 95% CI 0.89–1.29). A significant association of increased levels of Lp (a) with the risk of IS as compared to control groups (OR 1.54; 95% CI 1.21–1.86) was observed for case–control studies but not for prospective cohort studies (OR 2.23; 95% CI 0.92–3.54). High-quality studies confirmed a significant association of increased levels of Lp (a) with the risk of IS as compared to control groups (OR 1.98; 95% CI 1.69–2.26) but not the medium (OR 1.01; 95% CI 0.95–1.08) and low-quality studies (OR 2.40; 95% CI 0.40–4.40) (Fig. 3).

### Association of Lp (a) levels with the risk of large artery atherosclerosis

The association of Lp (a) levels with the risk of LAA stroke subtype vs. control was investigated in eight studies and our findings reveal an overall significant association of increased levels of Lp (a) with the risk of LAA as compared to control groups (SMD 0.32; 95% CI 0.00–0.64). Based on ethnicity, no significant association for the increased levels of Lp (a) with the risk of LAA in Asian population (SMD 0.08; 95% CI − 0.22 to 0.39) as well as Caucasian population (SMD 0.45; 95% CI − 0.10 to 0.64) was observed. All included eight studies were of case–control design which showed a significant association and based on NOS quality grading, no association was observed for medium quality studies (SMD 0.99; 95% CI − 0.23 to 0.74). Single studies were found based on high and low NOS quality and reported significant association for the increased levels of Lp (a) with the risk of LAA as compared to control groups (Table 4).

### Association of Lp (a) levels with the risk of small vessel disease

No significant association for the increased Lp (a) levels with the risk of SVD subtype vs. control (SMD − 0.06; 95% CI − 0.46 to 0.34) was observed. Moreover, based on ethnicity, study design and NOS quality grading, similar non-significant association was observed except for high quality study which included only a single study for SVD vs. control subjects (SMD 0.14; 95% CI 0.04 to 0.24) (Table 4).

### Association of Lp (a) levels with the risk of cardioembolic stroke

No significant association for the increased Lp (a) levels with the risk of CE stroke of IS subtype vs. control (SMD − 0.06; 95% CI − 0.46 to 0.34) was observed. Subgroup analysis based on ethnicity, study design and NOS quality grading also revealed a non-significant association for the increased Lp (a) levels with the risk of CE subtype vs. control subjects (Table 4).

### Association of Lp (a) levels with the risk of intracerebral hemorrhage

**Figure 4:**
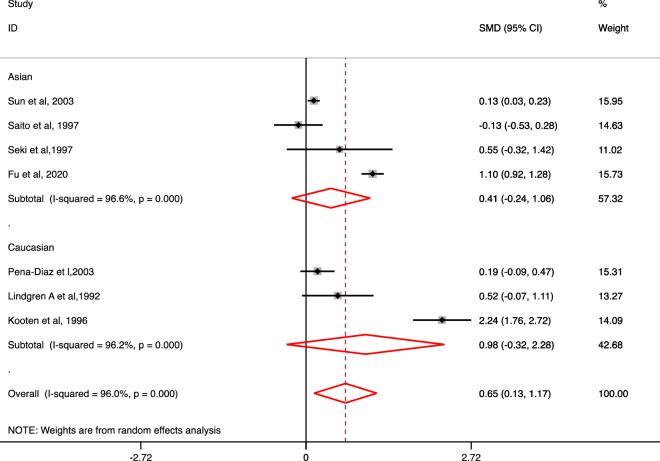
Forest plot for the association of Lp (a) level with the risk of Intracerebral hemorrhage (ICH) vs. control based on ethnicity.

Overall, a significant association for the association of increased Lp (a) levels with the risk of ICH as compared to control subjects (SMD 0.65; 95% CI 0.13–1.17) was observed (Fig. 4). Non-significant association for the increased Lp (a) levels with the risk of ICH vs. control was observed based on ethnicity and study design. However, after conducting a subgroup analysis based on NOS quality grading, a significant association for the increased Lp (a) levels with the risk of ICH as compared to control subjects (SMD 0.27; 95% CI 0.03–0.52) was only observed in medium quality studies (Table 4).

### Publication bias analysis

The shape of the funnel plots indicated the presence of publication bias while analysing the Lp (a) levels with the overall risk of IS. After conducting the Begg’s test, we observed that a significant publication bias was present in the included studies for Lp (a) levels with the overall risk of IS (p-value: 0.002) (Figure S-1a). The shape of other funnel plots for the included studies of IS subtypes and ICH in the meta-analysis did not indicate the presence of any publication bias (Figure S-1b–e).

### Meta-regression analysis

To further explore the amount of heterogeneity present in our meta-analysis, we conducted meta-regression analysis based on NOS quality score, study design and ethnicity for determining the impact of heterogeneity. Significant heterogeneity was observed for overall IS vs. control based on NOS quality (p = 0.005) (see Supplementary figure S-2a). We observed that NOS quality score and ethnicity was not associated with the overall effect size in any of the outcomes measured in the meta-analysis (Supplementary Figure S-2b–e, S-3 and S-4a–e). For LAA, SVD, CE and ICH, all studies were of case–control design, hence meta-regression was not possible for these groups based on study design.

### Sensitivity analysis

A sensitivity analysis was conducted by omitting a single study in each turn to determine if the overall effect size was influenced by the exclusion of a single study. Overall, no impact was observed for IS vs. control group. The sensitivity analysis suggested significant outliers for the included studies by two studies (Sun et al*.* 2003^45^ and Petersen et al*.* 2007^66^) investigating for the association of Lp (a) with LAA vs. control; two studies (Sun et al*.* 2003^45^ and Chakraborty et al*.* 2013^53^) for SVD vs. control; three studies (Kooten et al*.* 1996^64^ Chakraborty et al*.* 2013^53^ and Petersen et al*.* 2007^66^) for CE vs. control; three studies (Kooten et al*.* 1996^64^ Sun et al*.* 2003^45^ and Fu et al. 2020^38^) for ICH vs. Control which could have potentially affected the overall effect size estimates (Figure S-3a–e).

## Discussion

The present systematic review and meta-analysis of 45 studies analysed the potential role of Lp (a) levels and its association with the risk of IS, IS subtypes based on TOAST classification and ICH compared to control subjects. To the best of our knowledge, this is the most robust and the largest meta-analysis conducted till date which comprised of both Asian and Caucasian ethnicities to ascertain the risk of IS, IS subtypes and HS with increased levels of Lp (a). Our meta-analysis revealed that increased levels of Lp (a) are significantly associated with the risk of IS in Asian as well as Caucasian population. Also a significant association for the increased level of Lp (a) with the risk of LAA and ICH as compared to control subjects was observed. Lp (a) levels were found to be greater in Asian population as compared to Caucasian population confirming greater risk of stroke as compared to control group. However, on the basis of subtypes, no significant association was observed in Asian as well as Caucasian population separately for increased levels of Lp (a) in LAA, SVD and CE subtypes when compared to control groups.

Two previous meta-analyses had also established the association of elevated Lp (a) levels and risk of stroke by pooling data from case–control, prospective cohort and nested case–control studies^27,28^. A total of thirty-one studies were included in the meta-analysis by Smolders et al*.* 2007^27^ in which the association of overall stroke was found to be statistically significant with Lp (a) increment levels (SMD 0.39; 95% CI 0.23–0.54) which is in agreement with the findings of our meta-analysis. Another meta-analysis by Nave et al*.* 2015^28^ observed a significant association between Lp (a) and IS with OR of 1.41 (95% CI 1.26–1.57) for case–control studies (n = 11) and the pooled estimated risk ratio was 1.29 (95% CI 1.06–1.58) for prospective studies (n = 9).

Despite the fact that this systematic review and meta-analysis was undertaken comprehensively with defined inclusion and exclusion criteria along with uniform measured-effect across all analyses, the study has some following limitations: (1)included studies had a wide range of incorporated variables like age, ethnicity, sample size, study-design; (2) mean and standard deviations of Lp (a) levels obtained from few studies were converted from either the actual reported median values or the inter-quartile range values, inferring that they did not actually represent the original mean and standard deviation values of Lp (a) levels. (c) Subgroup analysis based on cut-off values of Lp (a) levels was not performed owing to non-availability of cut-off values of Lp (a) in majority of the included articles as represented in Tables 1, 2 and 3. (d) A random-effects model was used to account for the significant heterogeneity arising out of the studies. Therefore, large scale population based observational studies with defined clinical characteristics of the stroke-affected subjects and healthy controls are needed to ascertain the association of Lp (a) with either IS, subtypes of IS or ICH in a statistically significant manner.

## Conclusion

Increased Lp (a) levels could be considered as a predictive marker for identifying individuals who are at risk of developing IS, LAA and ICH.

## Supplementary Information

Supplementary Tables.

Supplementary Figures.
