# Supplementary material for: Lipoprotein (a) level as a risk factor for stroke and its subtype: A systematic review and meta-analysis
Source: Sci Rep. 2021 Aug 2;11:15660. doi: 10.1038/s41598-021-95141-0 (PMC8329213; doi:10.1038/s41598-021-95141-0)
Supplement: Supplementary file 2 — Supplementary Figures. [file 41598_2021_95141_MOESM2_ESM.doc]

**S-Figure-1 (a-e):** Begg’s Funnel Plot for the association of Lp (a) level with the risk of stroke types and subtypes vs. Control (a) Ischemic stroke vs. Control; (b) Large artery atherosclerosis vs. Control; (c) Small Vessel disease vs. Control; (d) Cardioembolic stroke vs. Control and (e) Intracerebral Hemorrhage vs. Control

| **(a)** **Ischemic stroke vs. Control** | **(b)Large artery atherosclerosis vs. Control** |
| --- | --- |
| **(c)** **Small vessel disease vs. Control** | **(d) Cardioembolic stroke vs. Control** |
| **(e)** **Intracerebral Hemorrhage vs. Control** | |

**S-Figure-2 (a-e):** Meta-regression plot for the association of Lp(a) level with the risk of stroke types and subtypes vs. Control (a) Ischemic stroke vs. Control; (b) Large artery atherosclerosis vs. Control; (c) Small Vessel disease vs. Control; (d) Cardioembolic stroke vs. Control and (e) Intracerebral Hemorrhage vs. Control based on NOS quality score

| **(a)** **Ischemic stroke vs. Control** | **(b)Large artery atherosclerosis vs. Control** |
| --- | --- |
| **(c)** **Small vessel disease vs. Control** | **(d) Cardioembolic stroke vs. Control** |
| **(e)** **Intracerebral Hemorrhage vs. Control** | |

**S-Figure-3:** Meta-regression plot for the association of Lp(a) level with the risk Ischemic stroke vs. Control based on study design

**S-Figure-4 (a-e):** Meta-regression plot for the association of Lp(a) level with the risk of stroke types and subtypes vs. Control (a) Ischemic stroke vs. Control; (b) Large artery atherosclerosis vs. Control; (c) Small Vessel disease vs. Control; (d) Cardioembolic stroke vs. Control and (e) Intracerebral Hemorrhage vs. Control based on ethnicity

| **(a)** **Ischemic stroke vs. Control** | **(b)Large artery atherosclerosis vs. Control** |
| --- | --- |
| **(c)** **Small vessel disease vs. Control** | **(d) Cardioembolic stroke vs. Control** |
| **(e)** **Intracerebral Hemorrhage vs. Control** | |

**S-Figure-5 (a-e):** Sensitivity Analysis for the association of Lp (a) level with the risk of of stroke types and subtypes vs. Control (a) Ischemic stroke vs. Control; (b) Large artery atherosclerosis vs. Control; (c) Small Vessel disease vs. Control; (d) Cardioembolic stroke vs. Control and (e) Intracerebral Hemorrhage vs. Control

**5 (a) Ischemic stroke vs. Control**

5 (b) Large artery atherosclerosis vs. Control

**
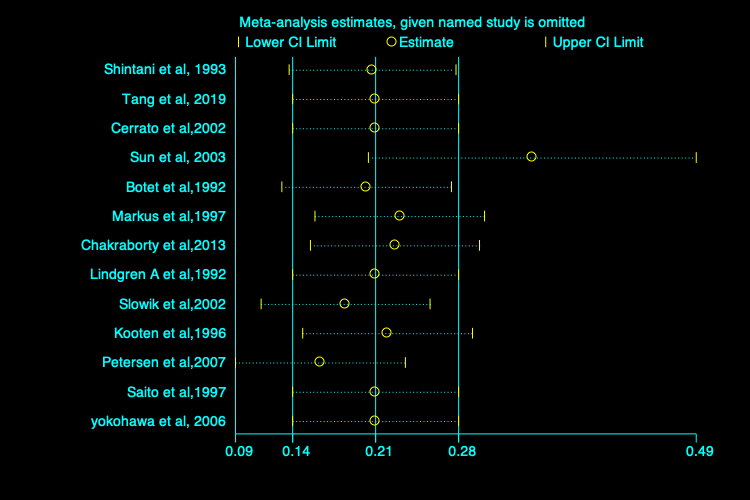
**

5(c) Small Vessel disease vs. Control


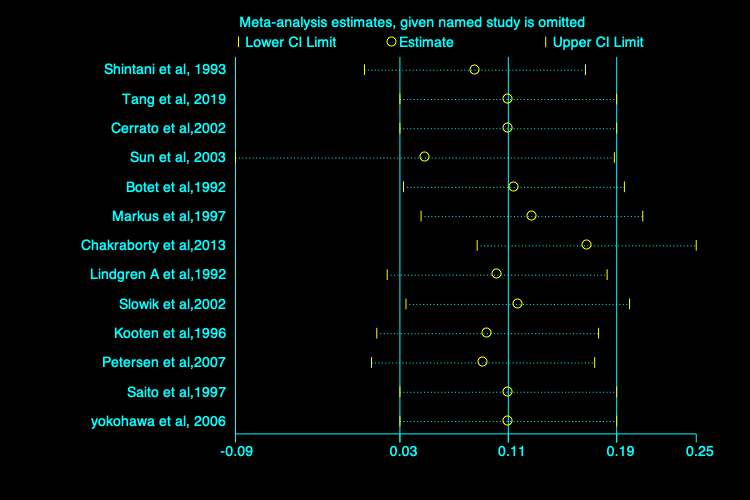


5 (d) Cardioembolic stroke vs. Control


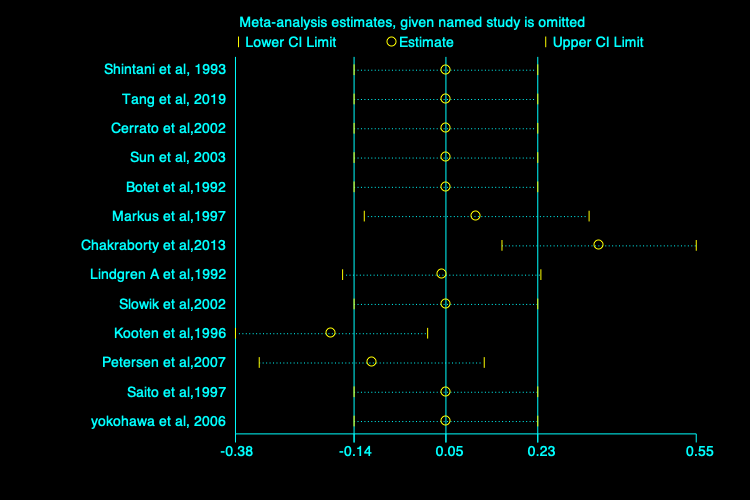


5 (e) Intracerebral Hemorrhage vs. Control

**
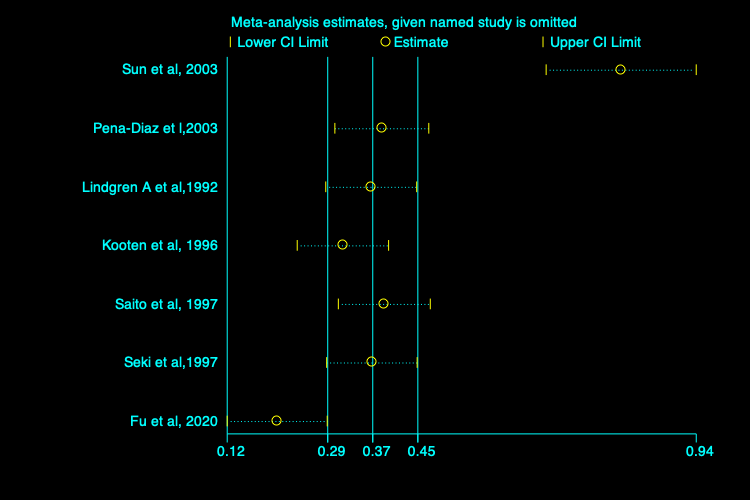
**
